# A Systematic Review Approach to Find Robust Items of the Zimbardo Time Perspective Inventory

**DOI:** 10.3389/fpsyg.2021.627578

**Published:** 2021-05-24

**Authors:** Chunhua Peng, Caizhen Yue, Andrew Avitt, Youguo Chen

**Affiliations:** ^1^Laboratory of Emotion and Mental Health, Chongqing University of Arts and Sciences, Chongqing, China; ^2^Collaborative Innovation Center for Brain Science, Chongqing, China; ^3^College of National Culture and Cognitive Science, Guizhou Minzu University, Guiyang, China; ^4^College of International Studies, Southwest University, Chongqing, China; ^5^Key Laboratory of Cognition and Personality of Ministry of Education, Time Psychology Research Center, Center of Studies for Psychology and Social Development, Faculty of Psychology, Southwest University, Chongqing, China

**Keywords:** time perspective, ZTPI, systematic review, psychometric problems, structural validity, internal consistency

## Abstract

The Zimbardo Time Perspective Inventory (ZTPI) is one of the most well-known and widely used measures of time perspective. Various short versions were proposed to resolve the psychometric problems of the ZTPI. The present study conducted a systematic review to obtain 25 short versions, calculated the frequency of each item of the ZTPI in short versions, and hypothesized that the more frequent the item is, the more robust it becomes. The hypothesis was tested by assessing the structural validity and internal consistency of short forms with high, medium, and low frequent items in Chinese samples (575 children, 407 undergraduates, and 411 older adults). Structural validity and internal consistency analyses showed that the form with more frequent items had better psychometric properties; item frequencies were positively correlated with factor loadings. The results suggest that the systematic review is an effective approach to identify the robust items of the ZTPI. This approach is general and can be the basis to improve the psychometric properties of scales in social science.

## Introduction

Time perspective is considered one of the most powerful influences on human behavior (Carstensen, 2006; Zimbardo et al., 2012). Time perspective originated from Lewin’s life space model, which included the influence of both the past and the future on current behavior. Time perspective corresponds to an individual’s view on his or her past and future at any given time (Lewin, 1942, 1951). Time perspective can be defined as the manner in which individuals partition the flow of their personal and social experiences into distinct temporal categories, which affects decision-making by locating the primary set of psychological influences within the temporal frames of either the present, past, or future (Zimbardo et al., 1997; Zimbardo and Boyd, 1999).

The Zimbardo Time Perspective Inventory (ZTPI) was developed to assess individual differences in time perspective (Zimbardo and Boyd, 1999). The scale was based on a conceptual model of the characteristic cognitive style and attitudes of those believed to be past-, present-, or future-oriented. The ZTPI measures time perspective in five factors: past negative (PN), past positive (PP), present fatalistic (PF), present hedonistic (PH), and future (F). PN reflects a negative or aversive attitude toward the past; PP represents a warm, sentimental, and nostalgic attitude toward their past; PH reflects an orientation toward present pleasure with little concern for future consequences; PF describes a helpless and hopeless belief about life; and F indicates behavior dominated by striving for future goals and rewards. The ZTPI has been translated into several languages, adapted in more than 20 countries and regions (Sircova et al., 2014), and cited more than 1,400 times in Scopus (Perry et al., 2020).

Previous studies reported mixed evidence regarding the psychometric properties of the ZTPI. The five-factor structure of time perspective not only has been replicated with exploratory factor analysis (EFA) in samples from France (Apostolidis and Fieulaine, 2004), Spain (Díaz-Morales, 2006), Romania (Cretu and Negovan-Zbăganu, 2013), as well as 23 countries (Sircova et al., 2014) but also has been confirmed with confirmatory factor analysis (CFA) in samples from the United States (Worrell and Mello, 2007), China (Wang et al., 2015), and Hungary (Orosz et al., 2017). However, several studies reported poor structural validity of the ZTPI, e.g., in a sample of 815 American adolescents (comparative fit index, CFI = 0.64) (Worrell and Mello, 2007), a sample of 476 American adults (CFI = 0.65) (Shipp et al., 2009), a sample of 247 Brazilian university students (CFI = 0.70) (Milfont et al., 2008), a sample of 419 Swedish adults (CFI = 0.63) (Carelli et al., 2011), and a sample of 303 Chinese university students (CFI = 0.48) (Wang et al., 2015). Previous studies also showed that internal consistency estimates for the ZTPI were not consistent. For example, Cronbach’s α of PP was below 0.70, and the α values of other subscales were above 0.70 (Worrell and Mello, 2007); the α values of all subscales were above 0.70 (Shipp et al., 2009); the α values of all subscales were below 0.70 (Milfont et al., 2008).

Several authors have attempted to overcome the limitations of the ZTPI by shortening the scale. Researchers proposed that short scales provide several important distinct advantages, such as reducing the fatigue of participants and better psychometric properties (Zhang et al., 2013; Orosz et al., 2017). Most of the short versions were developed based on samples in different countries, such as Greece (Anagnostopoulos and Griva, 2012), China (Chan et al., 2016), Romania (Cretu, 2012), and Germany (Danner et al., 2019) (Supplementary Table 1). However, previous research demonstrates that the factor structure and items of the short versions depend on the nationality of the sample (Przepiorka et al., 2016), that is, short scales usually have poor psychometric properties for samples independent from which they were developed (Temple et al., 2019).

A data-driven approach based on global data and a theory-driven approach were proposed to resolve the psychometric problems of the ZTPI. Sircova et al. (2014) assessed the structural equivalence of the ZTPI across 26 samples from 24 countries (*N* = 12,200). The study obtained a 36-item version of the ZTPI using EFA and CFA and found the five-factor structure of the ZTPI across 23 countries. The internal consistency and structural validity of the 36-item version of the ZTPI were examined in samples from the United Kingdom, the United States, and Australia, which provided support for the internal consistency, but revealed poor structural validity (McKay et al., 2015). Worrell et al. (2018) proposed a theory-driven approach to enhance the psychometric validity of the ZTPI, in which only items with a specific temporal content were retained (e.g., “past,” “tomorrow,” “future,” etc.). The study reported acceptable cross-cultural indexes for a new 25-item version of the ZTPI in the samples from the United Kingdom, the United States, Australia, and Slovenia. However, the CFA indexes were below the acceptable threshold for the short version from Worrell et al. (2018) study in samples from the United Kingdom, the United States, Australia, and Slovenia (CFI < 0.9, TLI < 0.9). Therefore, the data-driven approach based on global data (Sircova et al., 2014) and a theory-driven approach (Worrell et al., 2018) did not resolve the psychometric problems of the ZTPI satisfactorily.

Shortening the ZTPI is not an effective way to resolve the psychometric problems of the scale (McKay et al., 2015; Temple et al., 2019), and a new collaborative strategy is needed to address conceptual and measurement concerns with the ZTPI (Perry et al., 2020). As the first step, it is valuable to identify which item is “good” and which item is “bad” for the psychometric properties of the ZTPI. The present study aimed to converge the finding of previous short versions of the ZTPI and to identify the robust items of the ZTPI using a systematic review. The systematic review provides a method to combine findings from empirical studies using strict methodological requirements. Psychology can benefit from the systematic review because the systematic review summarizes the outcomes of many studies on a particular topic and identifies variables explaining differences (van Hemert, 2011; Furtado et al., 2019). Here, we firstly summarized short versions of the ZTPI using a systematic review and then calculated the frequency of each item appearing in the short versions. We hypothesized that the items with higher frequency are more robust to measure time perspective, that is, the short versions composed of more frequent items would have better psychometric properties. Finally, the hypothesis was tested in samples of Chinese adolescents and old adults. The aim of our studies was not to provide a new short version of the ZTPI but rather to provide a basis to improve the concept and measurement of time perspective in future work.

## Study 1

To obtain three short forms with high, medium, and low frequent items, we summarized the short versions of the ZTPI using a systematic review and calculated the frequency of items in the short versions.

### Materials and Methods

We performed the systematic review in accordance with the Preferred Reporting Items for Systematic Reviews and Meta-Analyses statement (Liberati et al., 2009). Published studies were identified by four research assistants on PsycINFO, PubMed, Web of Science, and Google Scholar. The last search was run on October 31, 2020. Search terms were “Zimbardo Time Perspective Inventory” and “ZTPI.”

English language studies reporting short versions of the ZTPI were included in the systematic review. We excluded studies in which the short versions included new items not in the original ZTPI (e.g., D’Alessio et al., 2003). In order to exclude potentially low-quality studies, only papers published in peer-reviewed journals were included.

### Results and Discussion

**FIGURE 1:**
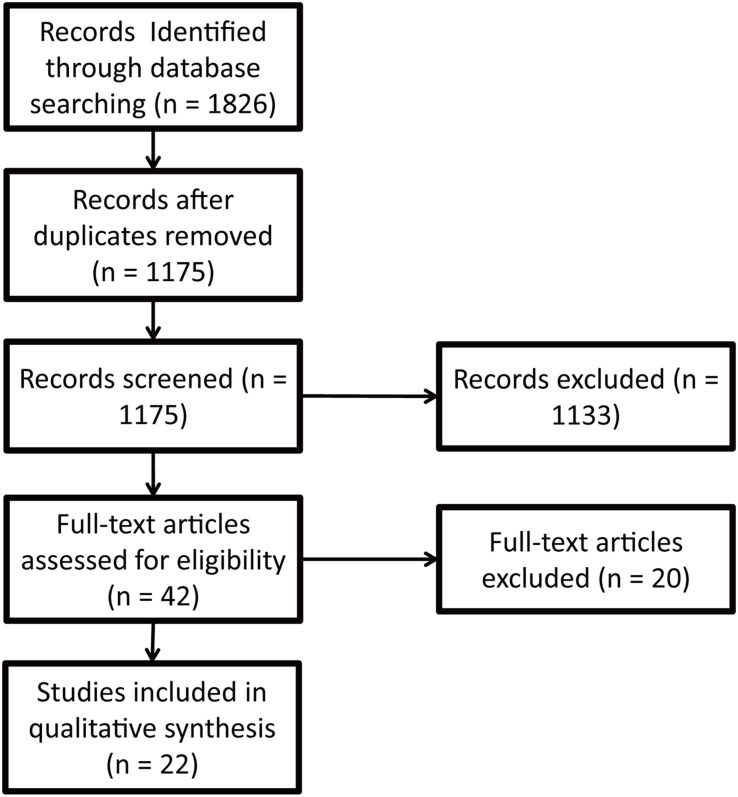
The procedure of study identification and selection.

The procedure of study identification and selection is illustrated in Figure 1. In total, 1,826 records were retrieved; 651 records were excluded due to duplications; 1,133 records were excluded because the studies were not in English, not related to the structure validity of the ZTPI, or not published in peer-reviewed journals. Forty-two full texts were checked, and 20 records were excluded because of no new short version or new short versions including new items not in the original ZTPI. Finally, 22 studies were identified for inclusion in the systematic review. Countries, participant age, structure factors, and items of 25 short versions of the ZTPI were presented in Supplementary Table 1. Short versions were developed in Greece, China, Romania, Germany, Chile, Latvia, Russia, Czech and Slovak Republics, Italy, Lithuania, Israel, Hungary, Japan, Poland, Estonia, Spain, Australia, the United Kingdom, the United States, and Slovenia. Participant ages ranged from 13 to 90 years. The number of structure factors ranged from three to six. We first counted the number of items in the Supplementary Table 1 and then calculated the frequency of each item. The frequency of an item is the ratio of the number of the item and the number of the short versions (Table 1).

**TABLE 1:** Frequency of each item of the ZTPI in 25 short versions and three short forms with high, medium, and low frequent items.

	Past negative	Past positive	Present hedonistic	Present fatalistic	Future
ZTPI-56	4 (0.60), 5 (0.16), 16 (0.76), 22 (0.64), 27 (0.52), 33 (0.32), 34 (0.80), 36 (0.56), 50 (1), 54 (0.56)	2 (0.76), 7 (0.72), 11 (0.60), 15 (0.52), 20 (0.84), 25 (0.48), 29 (0.60), 41 (0.16), 49 (0.44)	1 (0.28), 8 (0.52), 12 (0.20), 17 (0.48), 19 (0.32), 23 (0.48), 26 (0.72), 28 (0.44), 31 (0.80), 32 (0.44), 42 (0.80), 44 (0.48), 46 (0.44), 48 (0.44), 55 (0.36)	3 (0.40), 14 (0.72), 35 (0.32), 37 (0.84), 38 (0.76), 39 (0.84), 47 (0.40), 52 (0.32), 53 (0.40)	6 (0.36), 9 (0.24), 10 (0.72), 13 (0.84), 18 (0.36), 21 (0.72), 24 (0.40), 30 (0.56), 40 (0.88), 43 (0.40), 45 (0.84), 51 (0.48), 56 (0.32)
High	16, 34, 50	2, 7, 20	26, 31, 42	37, 38, 39	13, 21, 40, 45
Medium	4, 22, 54	11, 15, 29	8, 23, 44	14, 47, 53	10, 30, 43, 51
Low	5, 27, 33	25, 41, 49	1, 12, 19	3, 35, 52	6, 9, 18, 56

We obtained high, medium, and low frequent forms of the ZTPI based on the frequency of items (Table 1). Wang et al. (2015) revealed that the five-factor model had better fit indexes than the three-factor model (past, present, and future) in the Chinese context. Furthermore, Sircova et al. (2014) assessed the structural equivalence of the ZTPI across 26 samples from 24 countries and found the five-factor structure of the ZTPI across 23 countries (95.8%). Similarly, our systematic review showed that 19 out of 25 short versions included the five-factor structure (76%, Supplementary Table 1). Therefore, the three short forms include five factors the same as the original ZTPI. Besides, the number of items is equal for three short forms in one factor, and the frequencies of items in the high frequent form are all larger than 0.6. Thus, each short form has 16 items (Table 1).

## Study 2

To test the hypothesis that the items with higher frequency are more robust to measure time perspective, we assessed the psychometric properties of three short forms of the ZTPI based on the frequency of items in samples from Chinese children, undergraduates, and old adults.

### Materials and Methods

#### Participants

Data from three Chinese samples were analyzed. Participants in sample 1 consisted of 575 children from a middle school in Guangdong (aged 11–14, 45.7% female). Participants in sample 2 consisted of 407 undergraduates from two universities in Chongqing (aged 17–26, 64.6% female). Participants in sample 3 consisted of 411 adults in Chongqing (aged 62–94, 59.3% female). The study was conducted in accordance with the Declaration of Helsinki and was approved by the ethical board of the Southwest University.

#### Measures

The ZTPI contains 56 items (ZTPI-56). The ZTPI measures time perspective in five factors: PN, PP, PF, PH, and F (Zimbardo and Boyd, 1999). Participants were required to rate all items on a five-point Likert scale from 1 (very uncharacteristic) to 5 (very characteristic) according to their own situation. The Cronbach’s α ranges from 0.74 to 0.82 for each subscale (Zimbardo and Boyd, 1999). The Chinese version of the ZTPI was adapted from a Chinese translation of Zimbardo and Boyd (2010). The Chinese translation was translated back to English by a bilingual graduate student in English translation. A committee consisted of the graduate student, a bilingual professor, and a bilingual graduate student in psychology. The committee discussed discrepancies until they reached a consensus on a common version.

#### Procedure and Statistical Analyses

Time perspective was measured using the Chinese ZTPI for 575 children, 412 undergraduates, and 411 old adults. Determining sample size requirements for CFA remains a challenge, as the requirements are impacted by the number of factors and indicators, as well as the magnitude of factor loadings (Wolf et al., 2013). Researchers proposed several recommendations including a minimum sample size of 100, 200 (Boomsma, 1985), or 500 (Tabachnick and Fidell, 2013), and 5–20 cases per variable (Furr, 2018). To avoid the possible influence of insufficient sample size on conclusions, we separately analyzed the data from samples of children (sample size is 575, with about 10.3 cases per indicator for the ZTPI-56), undergraduates (407, with about 7.3 cases per indicator), and old adults (411, with about 7.4 cases per indicator), as well as the merged data (1,393, with about 24.9 cases per indicator).

R software with lavaan (Rosseel, 2012) and semTools (Jorgensen et al., 2020) was used for CFA. Ordinal variables were obtained with the five-point Likert scale. Previous studies reported that the Likert variables were not normally distributed (e.g., Flora and Curran, 2004; Li, 2016; Li et al., 2018). As maximum likelihood (ML) estimation assumes that the observed indicators follow a continuous and multivariate normal distribution, the ML is not appropriate for ordinal observed variables (Li, 2016). A WLSMV estimator is designed for ordinal data, which uses diagonally weighted least squares with robust variants to estimate the model parameters (Muthén, 1993; Rosseel, 2021). Thus, we used the WLSMV estimator to assess the ZTPI-56 and three short forms with high, medium, and low frequent items (Table 1). Since ML estimation was frequently used in previous studies, we also conducted a supplementary analysis using ML estimation (Supplementary Table 2). The Chi-square degree of freedom ratio (χ^2^/df), the CFI (comparative fit index), the TLI (Tucker Lewis index), and the RMSEA (root mean square error of approximation) and its 90% confidence interval (90% CI) were adopted to the criteria (Schreiber et al., 2006). We adopted Hu and Bentler’s (1999) recommended cutoffs: CFI and TLI greater than 0.95 and RMSEA below 0.06 as acceptable models.

### Results and Discussion

The structural validity of the ZTPI-56 and three short forms was assessed by conducting CFA on data from Chinese children, undergraduate, and old adult samples, as well as the merged data (Table 2). The high frequent form had the best fit indexes (CFI = 0.966–1.000, TLI = 0.957–1.002, RMSEA = 0.000–0.038), followed by the medium frequent form (CFI = 0.803–0.949, TLI = 0.748–0.935, RMSEA = 0.039–0.072) then the low frequent form (CFI = 0.659–0.909, TLI = 0.565–0.883, RMSEA = 0.038–0.078), and the worst was the ZTPI-56 (CFI = 0.634–0.829, TLI = 0.618–0.821, RMSEA = 0.060–0.081). Fit indexes of the low frequent form were not obtained for the merged data, which may be because the model was not identified. According to the cutoff values (CFI > 0.95, TLI > 0.95, RMSEA < 0.06), the high frequent form had acceptable fit indexes in three samples (Chinese children, undergraduates, and old adults) as well as the merged data.

**TABLE 2:** Fit indexes of CFA for ZTPI-56 and three short forms with high, medium, and low frequent items.

	χ ^2^	df	χ ^2^/df	CFI	TLI	RMSEA (90% CI)
Children						
ZTPI-56	5.377.321	1,474	3.648	0.771	0.760	0.068 (0.066, 0.070)
High	91.072	94	0.969	1.000	1.002	0.000 (0.000, 0.021)
Medium	226.940	94	2.414	0.923	0.901	0.050 (0.041, 0.058)
Low	199.816	94	2.126	0.882	0.850	0.044 (0.036, 0.053)
Undergraduates						
ZTPI-56	5.272.536	1.474	3.577	0.651	0.636	0.080 (0.077, 0.082)
High	109.151	94	1.161	0.986	0.982	0.020 (0.000, 0.034)
Medium	292.589	94	3.113	0.803	0.748	0.072 (0.063, 0.082)
Low	274.952	94	2.925	0.659	0.565	0.078 (0.068, 0.089)
Older adults						
ZTPI-56	3.680.770	1.474	2.497	0.829	0.821	0.060 (0.058, 0.063)
High	150.144	94	1.597	0.966	0.957	0.038 (0.026, 0.049)
Medium	153.434	94	1.632	0.949	0.935	0.039 (0.028, 0.050)
Low	150.042	94	1.596	0.909	0.883	0.038 (0.026, 0.049)
Merged data						
ZTPI-56	14,850.104	1.474	10.075	0.634	0.618	0.081 (0.080, 0.082)
High	244.496	94	2.601	0.967	0.958	0.034 (0.029, 0.039)
Medium	647.761	94	6.891	0.829	0.781	0.065 (0.060, 0.070)
Low*						

*lavaan WARNING: could not compute standard errors! The information matrix could not be inverted. This may be a symptom that the model is not identified.

The factor loadings of the items were obtained for the ZTPI-56 and three short forms in samples of Chinese children, undergraduates, and old adults, as well as the merged data. To further reveal the reason why the short form with higher frequent items had better fit indexes, the correlation analysis was performed on data from three Chinese samples as well as the merged data (Supplementary Figure 1). The item frequencies were positively correlated with the standardized factor loadings for the ZTPI-56 and three short forms in the samples of children, undergraduates, and old adults, as well as the merged data (*r* = 0.293–0.565, *p* values < 0.05).

The internal consistency of the ZTPI-56 and three short forms was assessed using Cronbach’s α and omega (Table 3). The ZTPI-56 had the best internal consistency (α = 0.619–0.770, ω = 0.620–0.778), followed by the high frequent form (α = 0.516–0.798, ω = 0.533–0.799), then the medium frequent form (α = 0.177–0.672, ω = 0.243–0.712), and the worst was the low frequent form (α = 0.062–0.443, ω = 0.001–0.444). Employing a value of 0.70 as acceptable, only 16 out of the 20 α coefficients and 15 out of the 20 ω coefficients were acceptable for the ZTPI-56; 8 out of the 20 α coefficients and 8 out of the 20 ω coefficients were acceptable for the high frequent form; no α coefficient and 1 out of the 20 ω coefficients was acceptable for the medium frequent form; no α coefficient and no ω coefficient was acceptable for the low frequent form.

**TABLE 3:** Cronbach’s α and ω estimates for ZTPI-56 and three short forms with high, medium, and low frequent items.

	Past negative		Past positive		Present hedonistic		Present fatalistic		Future	
	α	ω	α	ω	α	ω	α	ω	α	ω
Children										
ZTPI-56	0.735	0.745	0.735	0.738	0.735	0.720	0.714	0.718	0.770	0.778
High	0.762	0.762	0.691	0.699	0.798	0.799	0.583	0.589	0.629	0.640
Medium	0.370	0.401	0.578	0.605	0.668	0.712	0.451	0.464	0.549	0.561
Low	0.294	0.306	0.414	0.441	0.183	0.346	0.342	0.340	0.340	0.334
Undergraduates										
ZTPI-56	0.755	0.762	0.731	0.738	0.738	0.739	0.674	0.687	0.675	0.674
High	0.737	0.737	0.689	0.694	0.687	0.687	0.537	0.560	0.574	0.576
Medium	0.177	0.243	0.549	0.546	0.639	0.668	0.373	0.335	0.564	0.565
Low	0.443	0.444	0.347	0.377	0.190	0.140	0.184	0.193	0.132	0.004
Older adults										
ZTPI-56	0.723	0.733	0.727	0.757	0.746	0.756	0.619	0.620	0.658	0.636
High	0.656	0.658	0.767	0.782	0.783	0.785	0.516	0.533	0.572	0.575
Medium	0.421	0.434	0.672	0.674	0.536	0.635	0.296	0.313	0.535	0.542
Low	0.302	0.328	0.206	0.271	0.266	0.272	0.305	0.281	0.062	0.001
Merged data										
ZTPI-56	0.735	0.741	0.723	0.686	0.734	0.743	0.715	0.716	0.711	0.700
High	0.731	0.731	0.716	0.719	0.780	0.781	0.585	0.585	0.622	0.622
Medium	0.350	0.383	0.594	0.595	0.611	0.664	0.388	0.366	0.543	0.544
Low	0.338	0.335	0.272	0.237	0.258	0.285	0.344	0.336	0.219	0.092

## General Discussion

The purpose of these present studies was to identify the robust items of the ZTPI based on the systematic review. The structural validity and internal consistency of the ZTPI-56 and the high, medium, and low frequent forms were assessed in samples of Chinese children, undergraduates, and old adults. We found that the high frequent form had the best structural validity, followed by the medium frequent form, then the low frequent form, and the ZTPI-56; the item frequencies were positively correlated with the factor loadings; the ZTPI-56 had the best internal consistency, followed by the high frequent form, then the medium frequent form, and the low frequent form. The results supported our hypothesis that the items with higher frequency are more robust to measure time perspective.

The present study showed that the Chinese version of the ZTPI-56 was inadequate in structural validity. The CFI was from 0.63 to 0.83 for the Chinese version of the ZTPI-56 in samples of Chinese children, undergraduates, and old adults, as well as the merged data. This result was consistent with a sample of American adolescents (CFI = 0.64) (Worrell and Mello, 2007), a sample of American adults (CFI = 0.65) (Shipp et al., 2009), and a sample of Swedish adults (CFI = 0.63) (Carelli et al., 2011). Especially, using the ML estimation, we found that the CFI was 0.49 for the Chinese ZTPI-56 in a sample of Chinese undergraduates (Supplementary Table 2), which was almost the same with a previous study (CFI = 0.48) (Wang et al., 2015). Our study and Wang et al.’s (2015) study both adapted the Chinese ZTPI-56 from a Chinese translation of Zimbardo and Boyd (2010), but two studies conducted the adaptation independently. The above similar results suggested that the revision of the Chinese ZTPI-56 was appropriate in this study. Furthermore, we found that the structural validity of the ZTPI-56 was poorer than those of the short forms. This result was widely reported by previous studies, which is the reason why several authors attempt to resolve the psychometric problems of the ZTPI by shortening the scale (Wang et al., 2015; Orosz et al., 2017; Temple et al., 2019; Perry et al., 2020).

For the internal consistency of the Chinese ZTPI-56, Cronbach’s α was from 0.62 to 0.77, and ω was from 0.62 to 0.78. This result was consistent with the internal consistency of the ZTPI-56 in an American sample (α = 0.61–0.82), a British adolescent sample (α = 0.63–0.82), and a British university sample (α = 0.61–0.82) (Perry et al., 2020). Furthermore, we found that the internal consistency of the ZTPI-56 was better than those of the three short forms. The result was consistent with a previous finding that the lower the number of items are, the lower the Cronbach’s α will be (Cortina, 1993; Orosz et al., 2017). Thus, shortening the scale is not an ideal way to improve psychometric properties, especially for internal consistency.

A central finding of the present study was that the structural validity and internal consistency of short forms got better with an increase in item frequency (Tables 2, 3 and Supplementary Table 2). The frequency of each item was calculated based on 25 short versions, which were collected using a systematic review. We found that the factor loading increased as an increase in the item frequency for the ZTPI-56 and three short forms in samples of children, undergraduates, and old adults, as well as the merged data (Supplementary Figure 1). As factor loadings represent correlations between the indicators and the latent factors (Brown, 2015), the correlations between the indicators and the latent factors were stronger in the short form with higher frequent items. Thus, the short form with higher frequent items had better structural validity and internal consistency. Most of the short versions of the scale were created by data-driven approaches in specific samples, which improved the structural validity rather than the internal reliability, generalizability, and ability to detect individual differences in the construct (Perry et al., 2020). A theoretically driven, empirically tested approach could provide solutions for the above limitations. Worrell et al. (2018) reported that the short version including only explicit temporally phrased items had better structural validity and internal consistency compared with the ZTPI-56. The systematic review provides an effective way to integrate all the data-driven and theory-driven studies and provide information on which item is “good” and which item is “bad.” For example, item 50 is “I think about the bad things that have happened to me in the past” whose frequency is 1. Item 5 is “My decisions are mostly influenced by people and things around me” whose frequency is 0.16. Item 50 is “good,” and item 5 is “bad” to measure PN, which supports the Worrell et al. (2018) study that “good” items were accompanied by a specific temporal content (e.g., “past,” “tomorrow,” “future,” etc.).

One limitation of this study was that we cannot completely rule out the influence of cultures on the study. We hypothesized that the items with higher frequency are more robust to measure time perspective. The hypothesis is extracted from the systematic review. Twenty-five short versions were collected by the systematic review. The frequency of each item was calculated based on 25 short versions. The short versions were developed in samples from more than 20 countries, 17 out of 25 versions were developed in European samples, and only 6 out of 25 versions were developed in Asian, American, and Oceanian samples. Although Zimbardo’s five-factor structure of the time perspective was widely replicated in samples from different nations, such as France (Apostolidis and Fieulaine, 2004), Spain (Díaz-Morales, 2006), Japan (Pigott, 2018), and China (Wang et al., 2015), time perspective is also shaped by cultures (Jones and Brown, 2005). Hofstede and Bond (1988) found that people in nations with high Confucian dynamism (such as Thailand, China, Korea, and Japan) tend to be more hard work-, perseverance-, and future-oriented, while members of low Confucian dynamism cultures (such as Canada, Pakistan, and the United States) tend to be more past- and present-oriented. Therefore, cultural differences in the time perspective may lead to bias in the item selection. Furthermore, our hypothesis was only tested in the Chinese samples. Although the hypothesis is not specific to the Chinese sample, it still needs to be tested in samples from other countries.

## Conclusion

To sum up, the present study conducts a systematic review to calculate the frequency of items in 25 short versions of the ZTPI. The high, medium, and low frequent forms were developed based on the frequency of items. The psychometric properties of the three forms were assessed in Chinese samples. The results showed that the short form with higher frequent items yield more acceptable CFA results and stronger internal consistency estimates. The present study provided an approach to identify the “good” items and the “bad” items for psychometric properties, which would be the basis for further work to resolve the psychometric problems of scales.

## Data Availability Statement

The raw data supporting the conclusions of this article will be made available by the authors, without undue reservation.

## Ethics Statement

The studies involving human participants were reviewed and approved by the Ethics Committee of the Southwest University. The patients/participants provided their written informed consent to participate in this study.

## Author Contributions

YC designed the studies. CP and CY performed the studies. YC and CP analyzed the data. CP, YC, and AA wrote the manuscript. All authors approved the final manuscript as submitted and agreed to be accountable for all aspects of the work.

## Conflict of Interest

The authors declare that the research was conducted in the absence of any commercial or financial relationships that could be construed as a potential conflict of interest.

## Supplementary Material

Supplementary Figure 1 The correlations between item frequencies and factor loadings in samples of **(A)** Chinese children, **(B)** undergraduates, and **(C)** old adults, as well as **(D)** the merged data.

Supplementary Table 1 Structure factors and items of 25 short versions of the Zimbardo time perspective inventory (ZTPI).

Supplementary Table 2 Fit indexes of CFA with maximum likelihood estimation for the ZTPI-56 and three short forms with high, medium, and low frequent items.

Supplementary Table 3 Test of normality for Likert response of the ZTPI-56.

Supplementary Table 4 Fit indexes for multigroup CFAs testing measurement invariance of the five-factor structure of the high frequent form.
