# Supplementary material for: A Systematic Review Approach to Find Robust Items of the Zimbardo Time Perspective Inventory
Source: Front Psychol. 2021 May 24;12:627578. doi: 10.3389/fpsyg.2021.627578 (PMC8182797; doi:10.3389/fpsyg.2021.627578)
Supplement: Supplementary Figure 1 — The correlations between item frequencies and factor loadings in samples of (A) Chinese children, (B) undergraduates, and (C) old adults, as well as (D) the merged data. [file Table_1.docx]

Supplementary Material

**Supplementary Table 1.** Structure factors and items of 25 short versions of the Zimbardo time perspective inventory (ZPTI).

| Study | Country | Participants age | Sample size | Items | | | | | |
| --- | --- | --- | --- | --- | --- | --- | --- | --- | --- |
|  |  |  |  | Factor 1 | Factor 2 | Factor 3 | Factor 4 | Factor 5 | Factor 6 |
| (Anagnostopoulos and Griva, 2012) | Greece | 17–50 years | 457 | 4, 11, 16, 22, 25, 34, 36, 50, 54 | 2, 7, 15, 20, 29, 41, 49 | 1, 8, 12, 17, 19, 23, 26, 28, 31, 32, 42, 44, 46, 48, 55 | 3, 14, 37, 38, 39, 47, 53 | 6, 9, 10, 13, 18, 21, 24, 30, 40, 43, 45, 51, 56 |  |
| (Chan et al., 2016) | China | 12–19 years | 8,422 | 16, 22, 27, 33, 34, 36, 50, 54 | 2, 7, 11, 15, 20, 29 | 26, 28, 31, 32, 42 | 3, 14, 37, 38, 39 | 10, 13, 21, 30, 40, 43, 45, 51, 56 | 8, 23, 44, 46 |
| (Cretu, 2012) | Romania | 15–79 years | 1,260 | 16, 34, 50 | 2, 7, 20 | 31, 42 | 14, 37, 38, 39 | 13, 40, 45 |  |
| (Cretu and Negovan-Zbăganu, 2013) | Romania | 15–79 years | 1,260 | 16, 22, 27, 34, 36, 50 | 2, 7, 15, 20, 29 | 17, 26, 31, 42, 48, 55 | 3, 14, 35, 37, 38, 39, 53 | 10, 13, 18, 21, 30, 40, 45 |  |
| (Danner et al., 2019) | Germany | 18–70 years | 2,031 | 4, 50 | 11, 20 | 26, 31 | 14, 38 | 40, 45 |  |
| (Güell and Yopo, 2016) | Chile | 18–more than 65 years | 5, 008 | 4, 50 | 2, 20 | 32, 37, 39 | 21, 45 |  |  |
| (Kolesovs, 2009) | Latvia | M = 17.73, SD = 0.61 years | 278 | 11, 16, 22, 25, 34, 50 | 26, 44, 48, 46, 55 | 13, 21, 40 | 17, 19, 31, 42 | 14, 33, 37, 38, 39 | 6, 24, 30, 43 |
| (Kolesovs, 2009) | Russia | M = 17.65, SD = 0.60 years | 407 | 16, 22, 27, 34, 36, 50, 54 | 10, 13, 21, 30, 40, 51 | 26, 31, 42, | 6, 24, 43 | 37, 38, 39 | 28, 32, 45, 48, 52 |
| (Košťál et al., 2016) | Czech and Slovak Republics | 18–90 years | 2, 062 | 4, 50, 54 | 2, 7, 20 | 26, 42, 46 | 14, 38, 39 | 10, 40, 45 |  |
| (Laghi et al., 2013) | Italy | 17–19 years | 1, 300 | 16, 22, 25, 27, 50 | 2, 7, 11, 20, 29 | 17, 26, 28, 46, 48 | 3, 14, 37, 39, 53 | 10, 13, 21, 30, 45 |  |
| (Liniauskaitė and Kairys, 2009) | Lithuania | 18–85 years | 1, 244 | 4, 5, 16, 22, 25, 27, 33, 34, 36, 50, 54, | 2, 7, 11, 15, 20, 29, 41, 49 | 1, 8, 12, 17, 19, 23, 26, 31, 32, 42, 46, 48, 52, 55 | 3, 14, 24, 35, 37, 38, 39, 44, 47, 53 | 6, 9, 10, 13, 18, 21, 28, 30, 40, 43, 45, 51, 56 |  |
| (Orkibi, 2015) | Israel | 20–29 years | 572 | 16, 22, 34, 50 | 2, 20, 29, 49 | 1, 31, 42, 55 | 14, 37, 38, 39 | 10, 13, 45, 51 |  |
| (Orosz et al., 2017) | Hungary | 13–86 years | 1, 370 | 22, 25, 34, 50 | 15, 20, 29 | 31, 42, 46 | 37, 38, 39 | 13, 21, 40, 45 |  |
| (Pigott, 2018) | Japan | Not reported | 504 | 2, 4, 5, 16, 27, 34, 36, 50, 54 | 7, 11, 20, 22, 25, 29, 49 | 8, 26, 28, 31, 32, 42, 44, 55 | 3, 14, 37, 38, 39, 53 | 6, 10, 13, 18, 21, 24, 30, 40, 43, 45, 51, 52 |  |
| (Przepiorka et al., 2016) | Poland | 17–29 years | 805 | 4, 34, 36, 50, 54 | 2, 7, 11, 20, 49 | 8, 23, 31, 42, 44 | 10, 13, 21, 40, 45 |  |  |
| (Reuschenbach et al., 2013) | Germany | 15–85 years | 160 | 4, 9, 16, 22, 25, 27, 33, 34, 36, 50, 54 | 1, 2, 5, 7, 11, 15, 19, 20, 29, 41, 46, 49, 55 | 8, 12, 18, 21, 23, 26, 28, 30, 31, 32, 42, 44, 48, 52 | 3, 14, 28, 37, 39, 47, 53, 56 | 6, 10, 13, 17, 24, 35, 40, 43, 45, 51 |  |
| (Seema and Sircova, 2013) | Estonia | 18–54 years | 892 | 4, 16, 25, 34, 50 | 2, 7, 20, 29, 49 | 8, 17, 26, 31, 42 | 3, 14, 37, 38, 39 | 10, 13, 40, 45, 56 |  |
| (Sircova et al., 2014) | 24 countries | M = 26.9, SD = 12.3 years | 12, 200 | 4, 25, 27, 34, 36, 50, 54 | 2, 7, 11, 20, 29, 49 | 8, 12, 17, 19, 23, 31, 42, 44, 52, 55 | 24, 33, 35, 37, 38, 47 | 9, 10, 21, 30, 40, 45, 51 |  |
| (Sobol-Kwapinska et al., 2016) | Poland | 18–27 years | 877 | 4, 16, 22, 27, 33, 34, 36, 38, 47, 50, 54 | 1, 2, 7, 11, 15, 20 | 8, 17, 23, 26, 31, 42, 44, 46, 48 | 24, 28, 32, 35, 37, 39, 43, 52, 53 | 6, 13, 18, 21, 30, 40, 51 |  |
| (Sobol-Kwapinska et al., 2016) | Poland | 28–39 years | 448 | 4, 11, 16, 22, 25, 27, 33, 34, 35, 36, 37, 38, 47, 50, 54 | 7, 15, 20, 29, 49 | 1, 2, 17, 19, 26, 55 | 8, 9, 14, 23, 31, 32, 39, 41, 42, 44, 48, 52, 56 | 6, 10, 13, 18, 21, 30, 40, 43, 45, 51 |  |
| (Sobol-Kwapinska et al., 2016) | Poland | 40–65 years | 464 | 3, 4, 14, 16, 22, 27, 33, 34, 35, 36, 37, 38, 39, 47, 50, 54 | 2, 7, 10, 11, 13, 15, 17, 18, 20, 21, 26, 29, 30, 40, 45, 49, 51 | 8, 23, 28, 32, 42, 44, 48 |  |  |  |
| (Usart and Romero, 2014) | Spain | 18–57 years | 250 | 4, 16, 22, 25, 27, 29, 34, 36, 47, 50, 54 | 1, 2, 5, 7, 49 | 8, 19, 26, 31, 32, 42, 44, 46, 48 | 3, 9, 14, 15, 23, 24, 37, 38, 39, 43, 53 | 10, 13, 18, 21, 30, 35, 40, 45, 56 |  |
| (Wakefield et al., 2010) | Australia | 18–86 years | 614 | 4, 16, 34, 50, 54 | 7, 11, 15, 20, 29 | 8, 23, 26, 31, 42 | 14, 37, 38, 39, 53 | 10, 13, 40, 45, 51 |  |
| (Worrell et al., 2018) | UK, USA, Australia Slovenia | 11–70 years | 3, 261 | 16, 22, 27, 36, 50 | 2, 7, 11, 15, 25 | 12, 17, 19, 28, 46 | 37, 39, 47, 52 | 6, 13, 21, 24, 40, 56 |  |
| (Zhang et al., 2013) | USA | M = 27.0, SD = 10.6 years | 2, 225 | 16, 34, 50 | 2, 15, 20 | 23, 26, 31 | 14, 47, 53 | 10, 13, 40 |  |

**Supplementary Table 2.** Fit indexes of CFA with maximum likelihood estimation for ZTPI-56 and three short forms with high, medium, and low frequent items.

|  |  | ***χ^2^*** | ***df*** | ***χ^2^/df*** | **CFI** | **TLI** | **RMSEA[90%CI]** |
| --- | --- | --- | --- | --- | --- | --- | --- |
| Children | |  |  |  |  |  |  |
|  | ZTPI-56 | 4405.555 | 1474 | 2.989 | .626 | .609 | .059 [.057 .061] |
|  | **High** | **158.957** | **94** | **1.691** | **.966** | **.957** | **.035 [.025 .044]** |
|  | Medium | 251.839 | 94 | 2.679 | .885 | .853 | .054 [.046 .062] |
|  | Low | 253.794 | 94 | 2.700 | .770 | .707 | .054 [.046 .062] |
| Undergraduates | |  |  |  |  |  |  |
|  | ZTPI-56 | 4507.293 | 1474 | 3.058 | .492 | .469 | .071 [.069 .073] |
|  | High | 191.937 | 94 | 2.042 | .910 | .885 | .051 [.040 .061] |
|  | Medium | 344.241 | 94 | 3.662 | .724 | .648 | .081 [.072 .090] |
|  | Low | 309.175 | 94 | 3.289 | .573 | .455 | .075 [.066 .084] |
| Old adults | |  |  |  |  |  |  |
|  | ZTPI-56 | 3831.080 | 1474 | 2.599 | .576 | .557 | .062 [.060 .065] |
|  | High | 245.442 | 94 | 2.611 | .890 | .859 | .063 [.053 .072] |
|  | Medium | 208.540 | 94 | 2.219 | .871 | .835 | .054 [.045 .064] |
|  | Low | 205.667 | 94 | 2.188 | .760 | .694 | .054 [.044 .075] |
| Merged data | |  |  |  |  |  |  |
|  | ZTPI-56 | 9364.734 | 1474 | 6.353 | .634 | .618 | .081 [.080 .082] |
|  | High | 359.465 | 94 | 3.824 | .940 | .924 | .045 [.040 .050] |
|  | Medium | 779.198 | 94 | 8.289 | .773 | .710 | .072 [.068 .077] |
|  | Low* |  |  |  |  |  |  |

*lavaan WARNING: Could not compute standard errors! The information matrix could not be inverted. This may be a symptom that the model is not identified.

Fit indexes of the WLSMV estimation were better than that of the ML estimation, e.g. CFI = .771 – 1.000 for the WLSMV, and CFI = .626 – .957 for the ML in the sample of Children (supplementary Table 1). Kolmogorov-Smirnov and Shapiro-Wilk tests were conducted on Likert response of each item of the ZTPI with SPSS. Likert scale data were not normal for three samples (children, undergraduates, and old adults) as well as the merged data (p values < 0.001, supplementary Table 3). The results supported previous studies that the ML estimation obtained poorer fit indexes compared with the weighted least squares (WLS) for the nonnormal ordered categorical data (Hutchinson and Olmos, 1998; Flora and Curran, 2004; Muthen and Kaplan, 2011).

**Supplementary Table 3**. Test of normality for Likert response of the ZTPI-56.

| Item | Children | |  | Undergraduates | |  | Old adults | |
| --- | --- | --- | --- | --- | --- | --- | --- | --- |
|  | Kolmogorov-Smirnova | Shapiro-Wilk |  | Kolmogorov-Smirnova | Shapiro-Wilk |  | Kolmogorov-Smirnova | Shapiro-Wilk |
| 1 | 0.258*** | 0.835*** |  | 0.260*** | 0.803*** |  | 0.224*** | 0.835*** |
| 2 | 0.246*** | 0.825*** |  | 0.254*** | 0.831*** |  | 0.332*** | 0.735*** |
| 3 | 0.200*** | 0.910*** |  | 0.157*** | 0.909*** |  | 0.185*** | 0.909*** |
| 4 | 0.196*** | 0.908*** |  | 0.183*** | 0.910*** |  | 0.202*** | 0.883*** |
| 5 | 0.195*** | 0.913*** |  | 0.176*** | 0.901*** |  | 0.202*** | 0.913*** |
| 6 | 0.199*** | 0.908*** |  | 0.205*** | 0.893*** |  | 0.182*** | 0.896*** |
| 7 | 0.222*** | 0.889*** |  | 0.218*** | 0.873*** |  | 0.197*** | 0.865*** |
| 8 | 0.185*** | 0.913*** |  | 0.200*** | 0.882*** |  | 0.169*** | 0.916*** |
| 9 | 0.205*** | 0.907*** |  | 0.209*** | 0.883*** |  | 0.235*** | 0.828*** |
| 10 | 0.209*** | 0.891*** |  | 0.224*** | 0.900*** |  | 0.191*** | 0.888*** |
| 11 | 0.263*** | 0.870*** |  | 0.233*** | 0.874*** |  | 0.241*** | 0.815*** |
| 12 | 0.223*** | 0.900*** |  | 0.229*** | 0.878*** |  | 0.169*** | 0.889*** |
| 13 | 0.188*** | 0.907*** |  | 0.261*** | 0.881*** |  | 0.197*** | 0.880*** |
| 14 | 0.210*** | 0.904*** |  | 0.182*** | 0.900*** |  | 0.239*** | 0.821*** |
| 15 | 0.216*** | 0.898*** |  | 0.252*** | 0.873*** |  | 0.172*** | 0.901*** |
| 16 | 0.220*** | 0.904*** |  | 0.187*** | 0.896*** |  | 0.212*** | 0.894*** |
| 17 | 0.238*** | 0.882*** |  | 0.270*** | 0.825*** |  | 0.216*** | 0.848*** |
| 18 | 0.160*** | 0.917*** |  | 0.164*** | 0.903*** |  | 0.165*** | 0.899*** |
| 19 | 0.163*** | 0.897*** |  | 0.193*** | 0.880*** |  | 0.268*** | 0.794*** |
| 20 | 0.255*** | 0.880*** |  | 0.267*** | 0.847*** |  | 0.202*** | 0.865*** |
| 21 | 0.289*** | 0.850*** |  | 0.293*** | 0.817*** |  | 0.253*** | 0.834*** |
| 22 | 0.188*** | 0.886*** |  | 0.184*** | 0.880*** |  | 0.193*** | 0.867*** |
| 23 | 0.202*** | 0.904*** |  | 0.198*** | 0.873*** |  | 0.197*** | 0.901*** |
| 24 | 0.178*** | 0.915*** |  | 0.226*** | 0.882*** |  | 0.176*** | 0.907*** |
| 25 | 0.210*** | 0.877*** |  | 0.186*** | 0.883*** |  | 0.245*** | 0.825*** |
| 26 | 0.165*** | 0.916*** |  | 0.190*** | 0.885*** |  | 0.155*** | 0.911*** |
| 27 | 0.163*** | 0.917*** |  | 0.176*** | 0.892*** |  | 0.208*** | 0.869*** |
| 28 | 0.199*** | 0.905*** |  | 0.204*** | 0.896*** |  | 0.180*** | 0.901*** |
| 29 | 0.222*** | 0.891*** |  | 0.235*** | 0.838*** |  | 0.266*** | 0.806*** |
| 30 | 0.216*** | 0.897*** |  | 0.221*** | 0.894*** |  | 0.172*** | 0.899*** |
| 31 | 0.167*** | 0.916*** |  | 0.181*** | 0.895*** |  | 0.167*** | 0.904*** |
| 32 | 0.245*** | 0.887*** |  | 0.217*** | 0.883*** |  | 0.195*** | 0.865*** |
| 33 | 0.200*** | 0.901*** |  | 0.176*** | 0.914*** |  | 0.218*** | 0.897*** |
| 34 | 0.206*** | 0.909*** |  | 0.190*** | 0.900*** |  | 0.186*** | 0.903*** |
| 35 | 0.177*** | 0.914*** |  | 0.165*** | 0.914*** |  | 0.161*** | 0.911*** |
| 36 | 0.204*** | 0.906*** |  | 0.236*** | 0.893*** |  | 0.168*** | 0.912*** |
| 37 | 0.173*** | 0.915*** |  | 0.208*** | 0.905*** |  | 0.152*** | 0.913*** |
| 38 | 0.192*** | 0.911*** |  | 0.199*** | 0.901*** |  | 0.214*** | 0.870*** |
| 39 | 0.198*** | 0.908*** |  | 0.185*** | 0.891*** |  | 0.238*** | 0.812*** |
| 40 | 0.221*** | 0.893*** |  | 0.250*** | 0.878*** |  | 0.215*** | 0.886*** |
| 41 | 0.243*** | 0.882*** |  | 0.221*** | 0.870*** |  | 0.204*** | 0.874*** |
| 42 | 0.169*** | 0.914*** |  | 0.194*** | 0.889*** |  | 0.170*** | 0.912*** |
| 43 | 0.176*** | 0.915*** |  | 0.194*** | 0.897*** |  | 0.213*** | 0.870*** |
| 44 | 0.198*** | 0.912*** |  | 0.177*** | 0.914*** |  | 0.186*** | 0.913*** |
| 45 | 0.229*** | 0.895*** |  | 0.243*** | 0.889*** |  | 0.187*** | 0.905*** |
| 46 | 0.178*** | 0.915*** |  | 0.192*** | 0.896*** |  | 0.176*** | 0.913*** |
| 47 | 0.174*** | 0.915*** |  | 0.201*** | 0.881*** |  | 0.187*** | 0.898*** |
| 48 | 0.210*** | 0.900*** |  | 0.221*** | 0.894*** |  | 0.177*** | 0.886*** |
| 49 | 0.215*** | 0.893*** |  | 0.213*** | 0.863*** |  | 0.157*** | 0.896*** |
| 50 | 0.181*** | 0.914*** |  | 0.193*** | 0.900*** |  | 0.202*** | 0.905*** |
| 51 | 0.245*** | 0.889*** |  | 0.272*** | 0.861*** |  | 0.204*** | 0.890*** |
| 52 | 0.163*** | 0.917*** |  | 0.159*** | 0.901*** |  | 0.192*** | 0.880*** |
| 53 | 0.188*** | 0.908*** |  | 0.197*** | 0.902*** |  | 0.233*** | 0.854*** |
| 54 | 0.196*** | 0.906*** |  | 0.220*** | 0.896*** |  | 0.201*** | 0.892*** |
| 55 | 0.198*** | 0.908*** |  | 0.160*** | 0.911*** |  | 0.180*** | 0.897*** |
| 56 | 0.179*** | 0.911*** |  | 0.180*** | 0.908*** |  | 0.209*** | 0.893*** |

*** *p* < 0.001

Fit indexes of high frequent form were best in the sample of children (e.g. CFI =1.000), followed by the undergraduates (CFI = .986), and the old adults (CFI = .966, Table 1). To examine whether the five-factor model provided a different fit in each group for the high frequent form, we examined a measurement invariance across three samples with measurementInvariance command in semTools (Jorgensen et al., 2020). The WLSMV estimator was used to estimate parameters of multigroup confirmatory factor analyses (CFA) (Li and Liu, 2011). We tested three models: configural invariance (The five-factor structure was imposed on all groups), metric invariance (The factor loadings were constrained to be equal across groups), and scalar invariance (The factor loadings and intercepts were constrained to be equal across groups) (Di Giunta et al., 2017). We found full metric invariance across children, undergraduates, and old adults (supplementary Table 4). Thus we will not further discuss the age difference of the fit indexes.

**Supplementary Table 4**. Fit indexes for multigroup CFAs testing measurement invariance of the five-factor structure of the high frequent form.

|  | ***χ^2^*** | *df* | *CFI* | Δ***χ^2^*** | Δ*df* | *p* |
| --- | --- | --- | --- | --- | --- | --- |
| Configural invariance | 388.04 | 282 | 0.978 |  |  |  |
| Metric invariance | 445.88 | 304 | 0.970 | 27.499 | 22 | 0.193 |
| Scalar invariance | 578.4 | 326 | 0.947 | 109.425 | 22 | 0.000 |

**
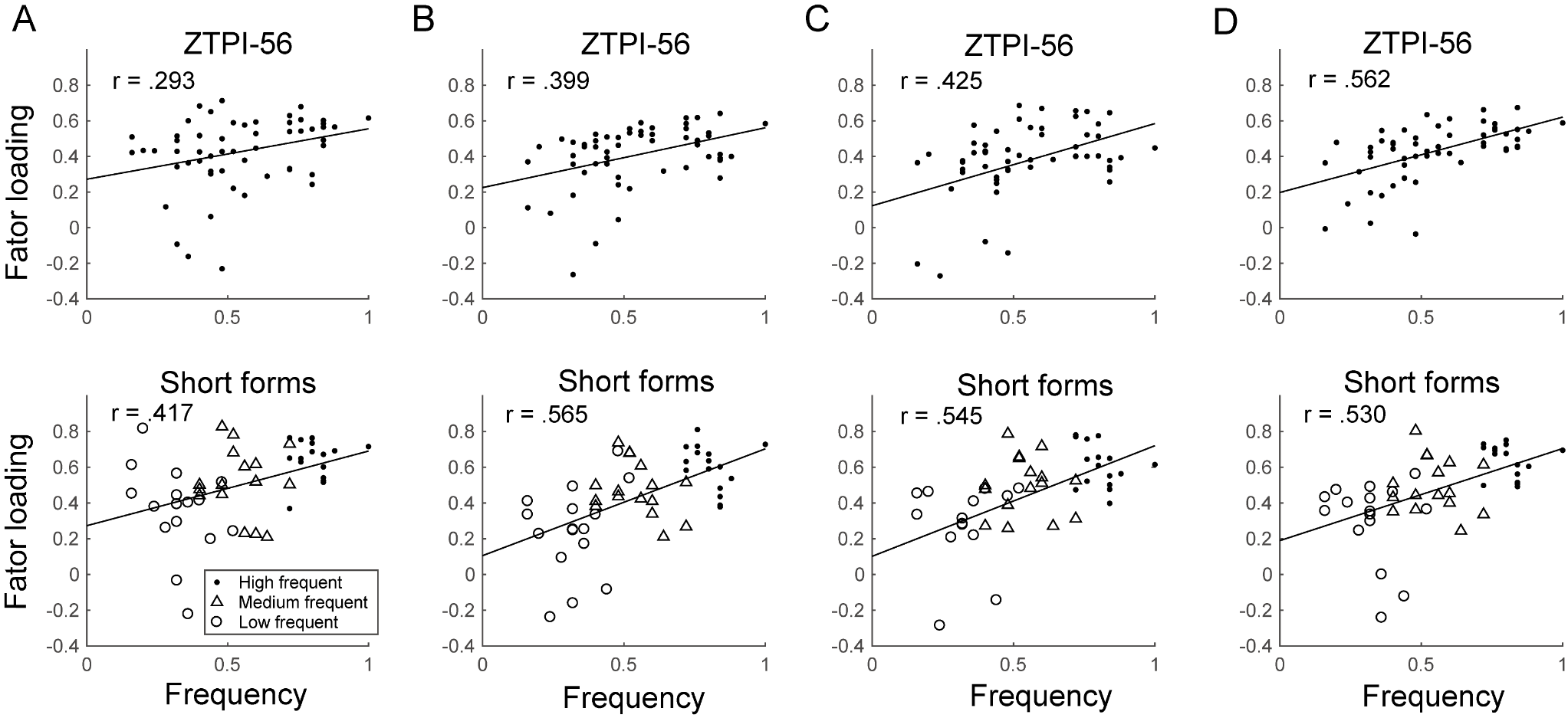
**

**Supplementary Figure 1**. The correlations between item frequencies and factor loadings in samples of (A) Chinese children, (B) undergraduates, and (C) old adults, as well as (D) the merged data.

**References**

Anagnostopoulos, F., and Griva, F. (2012). Exploring time perspective in Greek young adults: Validation of the Zimbardo Time Perspective Inventory and relationships with mental health indicators. *Soc. Indic. Res.* 106, 41–59. doi:10.1007/s11205-011-9792-y.

Chan, S. M., Kwok, W. W., and Fung, T. W. (2016). Psychometric properties of the Zimbardo Time Perspective Inventory in Hong Kong adolescents. *Time Soc.* 28, 33–49. doi:10.1177/0961463X16678250.

Cretu, R. Z. (2012). A confirmatory approach of the structure of Zimbardo’s time perspective concept. *Cogn. Brain Behav.* 16, 481–494.

Cretu, R. Z., and Negovan-Zbăganu, V. (2013). An exploratory approach of the structure of Zimbardo’s time perspective concept. *Procedia Soc. Behav. Sci.* 78, 753–757. doi:10.1016/j.sbspro.2013.04.389.

Danner, D., Treiber, L., and Bosnjak, M. (2019). Development and psychometric evaluation of a short version of the Time Perspective Inventory. *Eur. J. Psychol. Assess.* 35, 172–181. doi:10.1027/1015-5759/a000382.

Di Giunta, L., Iselin, A.-M. R., Eisenberg, N., Pastorelli, C., Gerbino, M., Lansford, J. E., et al. (2017). Measurement Invariance and Convergent Validity of Anger and Sadness Self-Regulation Among Youth From Six Cultural Groups. *Assessment* 24, 484–502. doi:10.1177/1073191115615214.

Flora, D. B., and Curran, P. J. (2004). An Empirical Evaluation of Alternative Methods of Estimation for Confirmatory Factor Analysis With Ordinal Data. *Psychol. Methods* 9, 466–491. doi:10.1037/1082-989X.9.4.466.

Güell, P., and Yopo, M. (2016). The subjective texture of time. An exploratory and empirical approach to time perspectives in Chile. *Time Soc.* 25, 295–319. doi:10.1177/0961463X15577260.

Hutchinson, S. R., and Olmos, A. (1998). Behavior of descriptive fit indexes in confirmatory factor analysis using ordered categorical data. *Struct. Equ. Modeling* 5, 344–364. doi:10.1080/10705519809540111.

Jorgensen, T. D., Pornprasertmanit, S., Schoemann, A. M., and Rosseel, Y. (2020). semTools: Useful tools for structural equation modeling. *R package version 0.5-3*. Available at: Retrieved from https://CRAN.R-project.org/package=semTools.

Kolesovs, A. (2009). Factorial validity of the Latvian and Russian versions of the Zimbardo Time Perspective Inventory in Latvia. *Baltic J. Psychol.* 10, 46–54.

Košťál, J., Klicperová-Baker, M., Lukavská, K., and Lukavský, J. (2016). Short version of the Zimbardo Time Perspective Inventory (ZTPI–short) with and without the Future-Negative scale, verified on nationally representative samples. *Time Soc.* 25, 169–192. doi:10.1177/0961463X15577254.

Laghi, F., Baiocco, R., Liga, F., Guarino, A., and Baumgartner, E. (2013). Identity status differences among Italian adolescents: Associations with time perspective. *Child. Youth Serv. Rev.* 35, 482–487. doi:10.1016/j.childyouth.2012.12.018.

Li, C., and Liu, H. Y. (2011). Simulation study of the detection of measurement invariance and its influential factors for ordinal categorical data. *Psychol. Sci. (In Chinese)* 34, 1482–1487. doi:10.16719/j.cnki.1671-6981.2011.06.037.

Liniauskaitė, A., and Kairys, A. (2009). The Lithuanian version of the Zimbardo Time Perspective Inventory (ZTPI). *Psichologija* 40, 66–87. doi:10.15388/Psichol.2009.0.2585.

Muthen, B., and Kaplan, D. (2011). A comparison of some methodologies for the factor analysis of non-normal Likert variables. *Br. J. Math. Stat. Psychol.* 38, 171–189. doi:10.1111/j.2044-8317.1985.tb00832.x.

Orkibi, H. (2015). Psychometric properties of the Hebrew short version of the Zimbardo Time Perspective Inventory. *Eval. Health Prof.* 38, 219–245. doi:10.1177/0163278714531601.

Orosz, G., Dombi, E., Tóth-Király, I., and Roland-Lévy, C. (2017). The less is more: The 17-item Zimbardo Time Perspective Inventory. *Curr. Psychol.* 36, 39–47. doi:10.1007/s12144-015-9382-2.

Pigott, J. (2018). A Zimbardo Time Perspective Inventory (ZTPI) survey of Japanese university students. *Int. J. Soc. Sci. Interdiscip. Stud*. 3, 55–66.

Przepiorka, A., Sobol-Kwapinska, M., and Jankowski, T. (2016). A polish short version of the Zimbardo Time Perspective Inventory. *Pers. Individ. Dif.* 101, 78–89. doi:10.1016/j.paid.2016.05.047.

Reuschenbach, B., Funke, J., Drevensek, A., and Ziegler, N. (2013). Testing a German version of the Zimbardo Time Perspective Inventory (ZTPI). *Annales Universitatis Paedagogicae Cracoviensis. Studia Psychologica* 6, 16–29.

Seema, R., and Sircova, A. (2013). Mindfulness–a time perspective? Estonian study. *Baltic J. Psychol.* 14, 4–21.

Sircova, A., Vijver, F. J. R. van de, Osin, E., Milfont, T. L., Fieulaine, N., Kislali-Erginbilgic, A., et al. (2014). A global look at time: A 24-country study of the equivalence of the Zimbardo Time Perspective Inventory. *SAGE Open* 4, 2158244013515686. doi:10.1177/2158244013515686.

Sobol-Kwapinska, M., Przepiorka, A., and Zimbardo, P. P. (2016). The structure of time perspective: Age-related differences in Poland: *Time Soc.* 28, 5–32. doi:10.1177/0961463X16656851.

Usart, M., and Romero, M. (2014). Spanish Zimbardo Time Perspective Inventory construction and validity among higher education students. *Rev. Electron. Investig. Psicoeduc. Psigopedag.* 12, 483–507.

Wakefield, C. E., Homewood, J., Taylor, A., Mahmut, M., and Meiser, B. (2010). Time perspective in hereditary cancer: psychometric properties of a short form of the Zimbardo Time Perspective Inventory in a community and clinical sample. *Genet. Test. Mol. Biomarkers* 14, 617–627. doi:10.1089/gtmb.2009.0185.

Worrell, F. C., Temple, E. C., McKay, M. T., Živkovič, U., Perry, J. L., Mello, Z. R., et al. (2018). A theoretical approach to resolving the psychometric problems associated with the Zimbardo Time Perspective Inventory: Results from the USA, Australia, Slovenia, and the United Kingdom. *Eur. J Psychol. Assess.* 34, 41–51. doi:10.1027/1015-5759/a000313.

Zhang, J. W., Howell, R. T., and Bowerman, T. (2013). Validating a brief measure of the Zimbardo Time Perspective Inventory. *Time Soc.* 22, 391–409. doi:10.1177/0961463X12441174.
